# Hydrodynamics-Based Transplacental Delivery as a Useful Noninvasive Tool for Manipulating Fetal Genome

**DOI:** 10.3390/cells9071744

**Published:** 2020-07-21

**Authors:** Shingo Nakamura, Naoko Ando, Satoshi Watanabe, Eri Akasaka, Masayuki Ishihara, Masahiro Sato

**Affiliations:** 1 Division of Biomedical Engineering, National Defense Medical College Research Institute, Saitama 359-8513, Japan; naoandokoro@gmail.com (N.A.); ishihara@ndmc.ac.jp (M.I.); 2 Animal Genome Unit, Institute of Livestock and Grassland Science, National Agriculture and Food Research Organization (NARO), Tsukuba, Ibaraki 305-0901, Japan; kettle@affrc.go.jp; 3 Section of Gene Expression Regulation, Frontier Science Research Center, Kagoshima University, Kagoshima 890-8544, Japan; stylistics777@yahoo.co.jp (E.A.); masasato@m.kufm.kagoshima-u.ac.jp (M.S.)

**Keywords:** cardiomyocytes, CRISPR/Cas9, fetal gene therapy, fetuses, genome editing, hydrodynamics-based gene delivery (HGD) system, myosin heavy-chain α, transplacental gene delivery (TPGD), TPGD for acquiring genome-edited fetuses (TPGD-GEF)

## Abstract

We previously demonstrated that the injection of pregnant wild-type female mice (carrying enhanced green fluorescent protein (EGFP)-expressing transgenic fetuses) at embryonic day (E) 12.5 with an all-in-one plasmid conferring the expression of both Cas9 and guide RNA (targeted to the *EGFP* cDNA) complexed with the gene delivery reagent, resulted in some fetuses exhibiting reduced fluorescence in their hearts and gene insertion/deletion (indel) mutations. In this study, we examined whether the endogenous myosin heavy-chain α (*MHCα*) gene can be successfully genome-edited by this method in the absence of a gene delivery reagent with potential fetal toxicity. For this, we employed a hydrodynamics-based gene delivery (HGD) system with the aim of ensuring fetal gene delivery rates and biosafety. We also investigated which embryonic stages are suitable for the induction of genome editing in fetuses. Of the three pregnant females injected at E9.5, one had mutated fetuses: all examined fetuses carried exogenous plasmid DNA, and four of 10 (40%) exhibited mosaic indel mutations in *MHCα*. Gene delivery to fetuses at E12.5 and E15.5 did not cause mutations. Thus, the HGD-based transplacental delivery of a genome editing vector may be able to manipulate the fetal genomes of E9.5 fetuses.

## 1. Introduction

In murine fetuses at mid-gestational stages (from embryonic day (E) 9.5 to E12.5; the day the vaginal plugs are found is designated as E0), there are many organ anlages, and organogenesis occurs actively [1,2,3,4,5,6]. To explore the molecular mechanism involved in early organogenesis and to generate animal models with defects in organogenesis, gene delivery of functional nucleic acids (NAs), such as plasmid DNA, RNA interference (as exemplified by siRNA), and genome editing components, to these fetuses is considered to be an important strategy [7]. Gene delivery targeted to mid-gestational fetuses is largely divided into two routes: one is in utero gene delivery, based on an injection of NAs into fetuses exposed externally with subsequent electroporation at the injected site, and the other is transplacental delivery of NAs complexed with DNA delivery reagents into pregnant females [8]. Since the former is performed by a local injection of NAs under a dissecting microscope, sites suitable for transfection appear to be very limited. Typically, neural stem cells located at the basal layer of the zona ventricle of the fetal brain have been successfully transfected [9]. In contrast, the latter is based on systemic transfection of fetal cells by NAs delivered via the blood stream after passing through the transplacental barrier; in this case, the NAs are complexed with DNA delivery reagents and administered through a tail vein injection [10].

Transplacental gene delivery, termed “TPGD” by our group [11], was first developed by Tsukamoto et al. [12], who found that transfection of early post-implantation embryos (E6.5) to mid-gestational fetuses (E12.5) is possible when plasmid DNA complexed with a transfection-promoting reagent is intravenously injected into pregnant female mice at E6.5 to E15.5. Since then, several researchers have reported that this system works well, although many of the results reported subsequently are different from those reported by Tsukamoto et al. [12]. For instance, Kikuchi et al. [13] demonstrated that TPGD performed at E12.5 resulted in the most efficient gene delivery to fetuses and that embryonic heart tissue is the most amenable to transfection by this method. Wu et al. [14] administered siRNA for the sex-determining region Y (*Sry*) gene to the pregnant female mice at E10.5 and found that the male-to-female sex reversal occurred in the treated female gonad. All of these experiments are based on the transient expression of a plasmid-based transgene (or oligonucleotide), thereby leading to the production of fetuses whose chromosomal structure remains intact. However, with the recent advent of gene editing technology, as exemplified by the clustered regularly interspaced short palindromic repeats (CRISPR)/Cas9 system, the transient expression of a plasmid carrying genome editing component is sufficient to cause mutations in target genes [15,16,17,18]. We intravenously introduced an all-in-one plasmid (called pCGSap1-*EGFP*), enabling the potential expression of both Cas9 and guide RNA (gRNA) targeted to enhanced green fluorescent protein (*EGFP*) cDNA in pregnant wild-type mice (who had been successfully mated with EGFP-expressing male transgenic mice) at E12.5. Molecular analysis of hearts showing reduced levels of EGFP revealed the presence of insertion/deletion (indel) mutations at the target locus (3/24 fetuses tested), although normal cells were also present [11]. These findings suggested that TPGD-based genome editing is functional in mid-gestational fetuses. We thus termed this technology “TPGD for acquiring genome-edited fetuses (TPGD-GEF)” [11]. 

In the case of TPGD using naked NAs, gene transfer to the fetus often fails, probably due to the presence of the blood-placental barrier (BPB) [19]. To ensure fetal gene delivery, many researchers have employed gene delivery reagents such as lipids, liposomes, and polyethylenimines (PEIs), which can all protect DNA from degradation, enable the DNA to pass through the BPB smoothly, and increase the transfection efficiency in fetal cells [10]. However, these gene delivery reagents are often toxic to fetuses and sometimes cause fetal malformation [20,21,22,23]. Considering the safety of the fetus, there is a need to develop TPGD without the use of such reagents.

Previously, we used TPGD-GEF to target *EGFP* cDNA (introduced exogenously into a mouse genome) using the lipid-based gene delivery reagent FuGENE6 (Promega KK, Tokyo, Japan) [11]. At this time, we had two questions: the first was whether it was possible to disrupt an endogenous gene by this technology, and the second was whether it was possible to perform TPGD-GEF in the absence of a gene delivery reagent, such as FuGENF6. In this study, we first aimed to disrupt an endogenous murine cardiac myosin heavy-chain α (*MHCα*) gene [24,25,26] using this technology. Mutations in the *MHCα* gene cause hypertrophic cardiomyopathy (HCM) and dilated cardiomyopathy (DCM) in humans [27]. Particularly, HCM is the leading cause of sudden death in young adults [28]. If it is possible to produce mice with mutated *MHCα* via TPGD-GEF, they will be useful models for developing therapeutic strategies for patients with HCM and DCM. We next employed a hydrodynamics-based gene delivery (HGD) system as the gene delivery reagent-free TPGD. HGD was developed to transfect cells of internal organs (especially hepatocytes) in vivo by administering a large amount of plasmid DNA-containing solution (> 2.5 mL per adult mouse) within a short period [29,30]. For HGD-based gene delivery, naked DNA is frequently used [29,30]; however, recently plasmid DNA dissolved in a TransIT-EE (Enhanced Expression) Hydrodynamic Delivery Solution (Takara Bio Inc., Kusatsu, Shiga, Japan; hereinafter referred to as TransIT-EE) has been often employed for in vivo gene delivery, including employment by our group [31,32]. TransIT-EE is a polyamine-based solution and no other gene delivery reagents are included. To our knowledge, the HGD-based delivery of genome editing components using TPGD has not yet been reported. Thus, hereinafter we term this technology (TPGD-GEF combined with HGD) “HGD-based TPGD-GEF”. In this study, we performed proof-of principle experiments to examine whether HGD-based TPGD-GEF enables the manipulation of fetal genomes.

## 2. Materials and Methods

### 2.1. Mice Used 

B6C3F1 (a hybrid between C57BL/6N and C3H/HeN) female mice (7-weeks old) were purchased from Japan SLC Inc. (Hamamatsu, Shizuoka, Japan) and served as recipients that were subjected to HGD-based TPGD-GEF. To obtain pregnant females, B6C3F1 female mice were naturally mated to C57BL/6 male mice (8–15 weeks old; purchased from Japan SLC Inc.). For all matings, 12:00 on the day the vaginal plugs were observed was designated as E0.5. All mice were maintained on a 12 h light/dark schedule (lights on from 07:00 to 19:00) and were allowed food and water ad libitum.

All animal experiments were performed at the National Defense Medical College (Tokorozawa, Saitama, Japan) in accordance with the guidelines of the National Defense Medical College Committee on Recombinant DNA Security, and approved by the Care and Use of Laboratory Animals (permission no. 16008; valid from 9 August 2016, to 31 March 2019, and no. 19007; valid from 23 July 2019, to 31 March 2020). All efforts were made to reduce the number of animals used and to minimize their suffering.

### 2.2. Construction of All-In-One Plasmid pCGSap1-MHCα Used for HGD-Based TPGD-GEF 

**Figure 1:**
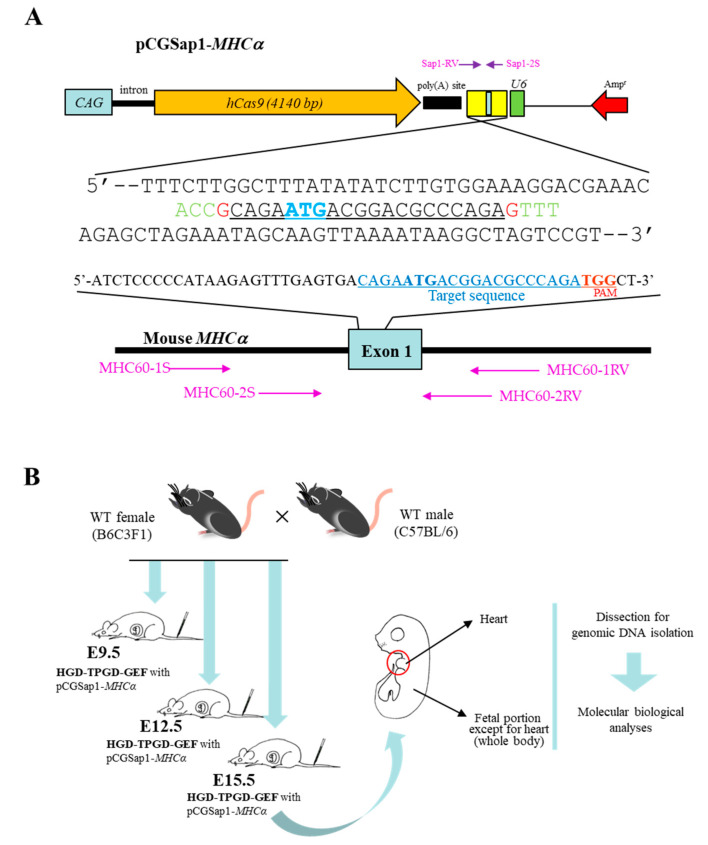
Plasmid used for hydrodynamics-based gene delivery (HGD)-based transplacental gene delivery for acquiring genome-edited fetuses (TPGD-GEF), and a flowchart for experiments using HGD-based TPGD-GEF. (**A**). Structure of pCGSap1 carrying a Cas9 expression unit (comprising *CAG*, the second intron of the rabbit β-globin gene, the humanized *Cas9* (*hCas9*) gene, and the poly(A) site of the rabbit β-globin gene) and a guide RNA (gRNA) expression unit (comprising *U6*, multiple sites into which chemically synthesized gRNA can be inserted, and a poly(A) site). As described in the Materials and Methods section, oligos targeting *MHCα* exon 1 were synthesized and inserted into the Sap I site of pCGSap1 to create pCGSap1-*MHCα*. The fidelity of the resultant pCGSap1-*MHCα* was confirmed by DNA sequencing using the Sap1-2S primer. The results of sequencing are shown at the bottom of **A**. The underlined sequence corresponds to the sequence of the gRNA targeting the *MHCα* gene. Nucleotides (shown in red and green) at both the 5′ and 3′ ends of the gRNA are those recognized by *Sap* I. The position of primers (Sap1-2S and Sap1-RV) used for identification of pCGSap1-*MHCα* are shown above the sequences. Furthermore, primers (MHC60-1S, -2S, -RV, and 2RV) used for amplification of exon 1 of *MHCα* are shown below the sequences. *CAG*, chicken β-actin based promoter; Amp^r^, ampicillin resistance gene; *U6*, human *U6* promoter. (**B**). Experimental flowchart for HGD-based TPGD-GEF. First, B6C3F1 females were mated to C57BL/6 male mice. On the day when females were confirmed to have copulatory plugs in their vaginas, 12:00 was defined as E (embryonic day) 0.5 of pregnancy. On E9.5, E12.5 or E15.5 of pregnancy, a TransIT-EE-based solution containing pCGSap1-*MHCα* was intravenously administered to the pregnant females. Two days after HGD, fetuses were dissected into fetal heart and the other parts designated as “whole body”. These samples were then subjected to molecular biological analyses for detection of pCGSap1-*MHCα* and mutations at the *MHCα* locus. WT, wild-type.

Two 25-mer oligonucleotides (oligos) (E-oligo-S/E-oligo-AS; shown in Table 1) targeting the upper portion of *MHCα* gene (Gene ID in the National Center for Biotechnology Information: 17888) were synthesized and subjected to annealing at 95 °C for 5 min followed by incubation at room temperature (~24 °C) for 30 min. The annealed oligo was then cloned into the *Sap* I site of the pCGSap1 vector (upper portion of Figure 1A) [33] to create pCGSap1-*MHCα.* The resultant plasmid should confer simultaneous expression of the humanized *Cas9* gene under the chicken β-actin-based promoter (CAG) and gRNA (targeted to *MHCα*) under the *U6* promoter, upon transfection. The constructed pCGSap1-*MHCα* was subjected to DNA sequencing to confirm that the introduced oligo had been correctly inserted into pCGSap1 (lower portion of Figure 1A).

### 2.3. HGD-Based TPGD-GEF and Isolation of Fetal Tissues

For HGD-based TPGD-GEF, we employed HGD as previously reported [31,32,34]. In brief, mice were injected with a 20 µg of plasmid DNA (pCGSap1-*MHCα*) containing TransIT-EE (one-tenth of the weight/volume (in mL) per mouse; for example, 3 mL/30 g of a mouse) using a syringe (3 mL Luer lock type; Nipro, Inc., Osaka, Japan) fitted with a 30-gauge needle (Dentronics Co., Ltd., Tokyo, Japan). Injections were performed at a constant injection speed via the tail vein and completed within 10 seconds by the same researcher in order to avoid artefactual effects in each experiment. Pregnant B6C3F1 females at E9.5, E12.5, or E15.5 of gestation were subjected to HGD-based TPGD-GEF. Control fetuses were obtained from B6C3F1 females receiving intravenous injection of TransIT-EE solution alone at E9.5 of gestation (mock injection). 

Prior to the injection of the DNA-containing solutions, mice were subjected to sufficient anesthesia by intraperitoneal (IP) injection of three combined anesthetics (medetomidine (0.75 mg/kg; Nippon Zenyaku Kogyo Co. Ltd., Koriyama, Fukushima, Japan), midazolam (4 mg/kg; Sandoz K.K., Tokyo, Japan), and butorphanol (5 mg/kg; Meiji Seika Pharma Co., Ltd., Tokyo, Japan). After the intravenous injection, the anesthetized mice were recovered by a subcutaneous injection of atipamezole (3.75 mg/kg; Nippon Zenyaku Kogyo Co. Ltd.), a medetomidine antagonist, and then maintained on an electric plate warmer for recovery from anesthesia. 

Two days after the injection, the female mice were euthanized, and their fetuses were isolated. The fetuses were separated from the yolk sacs and the hearts were dissected under a dissecting stereomicroscope (SMZ800; Nikon Co., Tokyo, Japan) and transferred to 1.5-mL tubes (#3810X; Eppendorf AG, Hamburg, Germany). The other fetal tissues, which are referred to hereinafter as “whole body”, were also transferred to tubes for genomic DNA isolation.

Genomic DNA was extracted by adding 600 μL of lysis buffer (0.06 mg/mL of proteinase K, 0.2 mg/mL of Pronase E, 10 mM Tris–HCl (pH 8.0), 100 mM NaCl, 10 mM EDTA (pH 8.0), 0.5% SDS) to the fetal tissue-containing tubes and then was subsequently incubated at 37 °C for 2 days with gentle shaking, followed by extraction with 100 μL saturated phenol [35]. The supernatant was precipitated with isopropanol, and the precipitated DNA was then dissolved in 100 μL of sterile water. The extracted genomic DNA was stored at 4 °C.

### 2.4. Molecular Biological Analysis Using PCR, Sub-Cloning, and Direct Sequencing 

The amplification reactions by PCR were performed in a total volume of 20 μL containing 10 mM Tris–HCl (pH 8.3); 50 mM KCl; 1.5 mM MgCl2; 0.25 mM each of dATP, dCTP, dGTP, and dTTP; 1 mM primers; ~ 5 ng of genomic DNA; 0.5 units of Taq polymerase (#R001A; Takara Bio Inc.) with each of the following primer sets (Table 1): (i) the Sap1-2S/Sap1-RV primer set was expected to yield 435-bp amplification products from the pCGSap1-*MHCα* plasmid, and was used for detection of the pCGSap1-*MHCα* transgene (Figure 1A); (ii) the mEx4-S/mEx4-RV primer set was expected to yield 390-bp products, and was used for the detection of the endogenous mouse α-1,3-galactosyltransferase gene (*GGTA1*) as a reference [11]; (iii) the MHC60-1S/MHC60-1RV primer set (Table 1; Figure 1A) was expected to yield 363-bp products, and was used for amplification of part of the endogenous *MHCα* gene (exon 1), spanning a region recognized by gRNA. In some cases, the MHC60-2S/MHC60-2RV primer set (Table 1; Figure 1A) was used for the nested PCR. This PCR was expected to generate 206-bp products.

Reactions of PCR were performed in an Applied Biosystems Veriti Thermal Cycler, Thermo Fisher Scientific K.K., Tokyo, Japan, with the following cycling conditions: 95 °C for 4 min followed by 95 °C for 30 s, 60 °C (65 °C in the case of PCR using the Sap1-2S/Sap1-RV primer set) for 30 s, 72 °C for 30 s for 35 cycles, and 72 °C for 4 min. For the negative controls, ~ 5 ng of genomic DNA from B6C3F1 fetal samples from mock-injected mice were used. Five nanograms of plasmid DNA (pCGSap1-*MHCα*) was concomitantly subjected to PCR as a positive control. For nested PCR, 2 μL of the first-round PCR product was subjected to second-round PCR in a 20 μL volume. The reaction components and their concentrations were the same as for the first-round PCR. Four microliters of each of the resulting PCR products was separated on a 2% agarose gel and stained with ethidium bromide (EtBr) for DNA visualization. 

Direct DNA sequencing was performed by Eurofins Genomics (Eurofins Genomics K.K., Tokyo, Japan) using the purified PCR products and MHC60-2S primer (Table 1; Figure 1A). Some of the PCR products that, upon direct sequencing, were found to have overlapping sequences, were sub-cloned into the TA cloning vector pCR2.1 (Invitrogen Co., Carlsbad, CA, USA). After selection via blue-white screening, sequencing of the plasmid DNA isolated from the white colonies was conducted.

### 2.5. Off-Target Analysis

Potential off-target sites were identified based on Cas-OFFinder program outputs (CRISPR RGEN Tools, Hanyang University, Korea, http://www.rgenome.net/cas-offinder/). From candidates with the highest scores, two genes were selected. In order to amplify these genes, genomic DNA (~0.5 ng) derived from fetus #a-1, -3, -4, and -7, all of which had been identified as having indel mutations, was subjected to PCR using different primer sets (Table 1) in a reaction volume of 20 μL, and employing the same PCR conditions described above. After purification of the resulting products, these samples were subjected to direct DNA sequencing using each specific sense-stand primer used for PCR.

## 3. Results

### 3.1. Timing of HGD-Based TPGD-GEF

In our previous study, based on the usual (non-HGD-based) method, we introduced plasmid DNA (pCGSap1-*EGFP*) complexed with FuGENE6 into the tail vein of pregnant B6C3F1 mice (which had been mated to a transgenic male) at E12.5. In this case, the method used for the DNA injection was based on HGD using pregnant B6C3F1 mice (which had been mated to wild-type C57BL/6 male mice) to target an endogenous gene (*MHCα*). Furthermore, instead of using FuGENE6, we employed TransIT-EE, which has been formulated as being adapted to HGD. To identify which embryonic stages were amenable to HGD-based TPGD-GEF, injections were performed on E9.5, E12.5, or E15.5.

**Figure 2:**
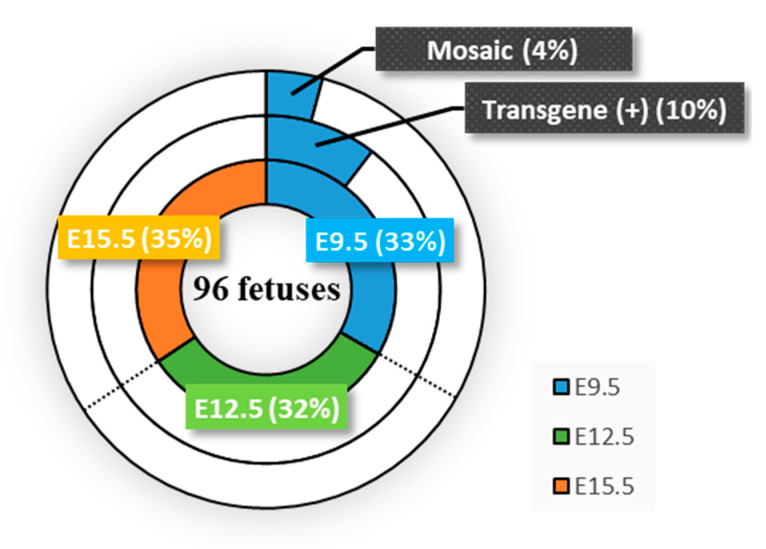
Summary of data on fetuses obtained after hydrodynamics-based gene delivery (HGD)-based transplacental gene delivery for acquiring genome-edited fetuses (TPGD-GEF) at E9.5, E12.5, and E15.5. Blue, green, and orange in the pie chart indicate the data obtained after HGD-based TPGD-GEF at E9.5, E12.5, and E15.5, respectively. A total of 96 fetuses were collected from the experiments and 10% of these fetuses were identified as having the plasmid DNA introduced. Finally, 4% of fetuses obtained exhibited successful genome editing at a target locus, although the mode was mosaic mutations. Notably, fetuses with transgenes and the genome-edited *MHCα* gene were all derived from HGD-based TPGD-GEF at E9.5.

A total of three females were used for each stage in the present study. Two days after HGD, fetuses were dissected and the fetal heart and other tissues (whole body) were separated from each other under a dissecting microscope, since, in our previous study, preferential expression of the introduced transgenes (coding for lacZ (β-galactosidase), with expression driven by the CAG promoter) was noted in the fetal hearts [13]. PCR-based genotyping of the dissected hearts using Sap1-2S/Sap1-RV primers (Table 1) revealed that of the 96 fetuses isolated from the nine pregnant females, 10 (10.4%) were found to have the pCGSap1-*MHCα* vector (Figure 2; Table S1; Figure S1A). These samples (termed #a-1 to -10) were all derived from female #A, who had been subjected to HGD at E9.5. However, there were no transgenic (Tg) fetuses from females #B and #C, who had been similarly injected on E9.5 (Figure 2; Table S1). Furthermore, no Tg fetuses were obtained from the pregnant females (three tested for each group) when HGD was performed on E12.5 and E15.5 (Figure 2; Table S1). Notably, the whole bodies in the same samples (#a-1 to -10) had the transgene in their genomes (Figure S1B). This finding suggested that the HGD-based TPGD-GEF on E9.5 enables the delivery of a transgene to all the cells in an animal’s body. 

### 3.2. Analysis of HGD-Based TPGD-GEF

**Figure 3:**
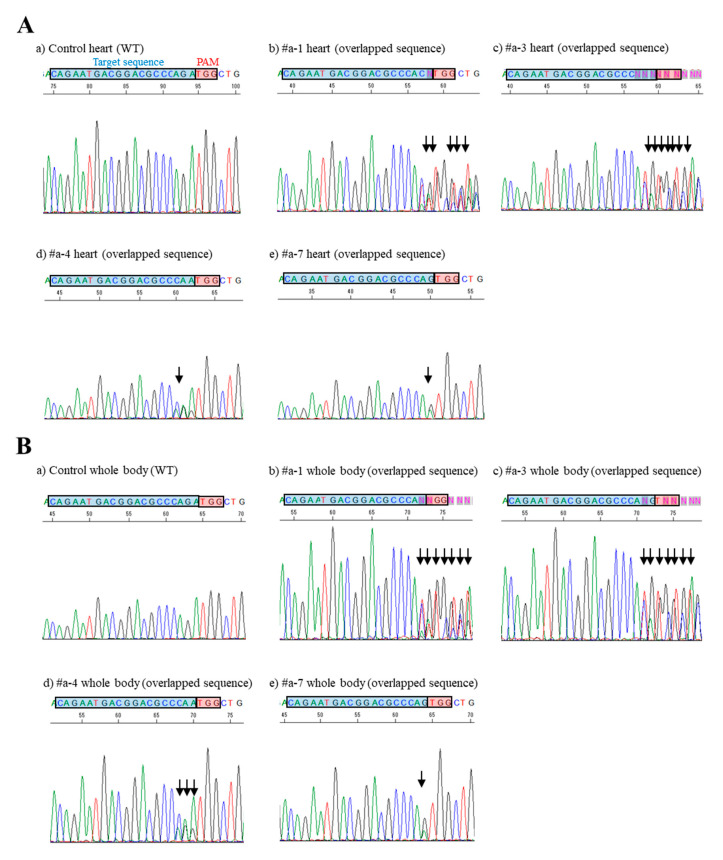
Sequence analysis of PCR products (corresponding to the endogenous *MHCα* gene exon 1) derived from HGD-based TPGD-GEF-treated fetuses. (**A**) Direct sequencing of PCR products obtained from fetal hearts (WT and #a-1, -3, -4, and -7). In a) control heart, the sequence enclosed by a quadrant filled with blue (corresponding to the target sequence recognized by gRNA targeting *MHCα* sequence) remained unaltered. By contrast, in b) #a-1, c) #a-3, d) #a-4, and e) #a-7, overlapping electrophoretograms (indicated by arrow) are notable immediately upstream of the PAM, indicating the presence of genome-edited and unedited sequences. The PAM (TGG) is shown as a quadrant filled with red. WT, wild-type. (**B**) Direct sequencing of PCR products obtained from murine fetal whole bodies (fetal portion except for heart) (WT and #a-1, -3, -4, and -7). There is a similar pattern of electrophoretograms as shown in **A**.

We next performed direct DNA sequencing of PCR-amplified products spanning a region (exon 1 of *MHCα* gene) containing the gRNA-binding target sequence using an MHC60-1S/MHC60-1RV primer set (Figure 1A; Table 1). The samples subjected to sequencing were those of the fetal cardiac samples (including #a-1 to -10 samples that had been judged positive for the presence of the transgene) as well as those (including #b-1 to -14, #c-1 to -8, #d-1 to -11, #e-1 to -11, #f-1 to -9, #g-1 to -12, #h-1 to -4, and #i-1 to -17) that were judged negative for the presence of the transgene (Figure 2; Table S1). Direct DNA sequencing demonstrated that of the 10 Tg samples examined, four (40%) had an overlapping electrophoretogram just upstream of the protospacer adjacent motif (PAM), suggesting a mixture of sequences derived from both genome-edited and unedited cardiac cells, which has been referred to as “mosaic” mutations (Figure 2; Figure 3A; Table S1). The other six Tg samples remained unaltered (Figure 2; Table S1). Non-Tg fetal samples had no *MHCα* indel mutations (Figure 2; Table S1). We also checked whether mutations involving the *MHCα* locus in samples #a-1, -3, -4, and -7, which had overlapping electrophoretograms, were detectable in the whole body samples. As shown in Figure 3B, similar patterns of overlapping electrophoretograms were also seen in these whole body samples. 

To examine the results of the DNA sequence analysis shown in Figure 3 in more detail, the PCR products of fetal cardiac samples #a-1, -3, -4, and -7 were sub-cloned into a pTA cloning vector. After selection via blue–white colony screening, DNA sequencing of the plasmids isolated from the white colonies using a primer MHC60-1S (in some cases, MHC60-2S) demonstrated that of the 12 #a-1-derived clones examined, three clones (25%) had a 1-bp (G) deletion between the third and first nucleotides upstream of the PAM; two clones (17%) had a 2-bp (AG or GA) deletion adjacent to the PAM, or between the fourth and first nucleotides upstream of the PAM; one clone (8%) had a 1-bp (G to C) replacement at the second nucleotide flanking the PAM (Table S2; Figure S2A-a). Six of the 12 clones (50%) examined had a normal sequence (Table S2). Sequencing of PCR products derived from the whole body sample of #a-1 also demonstrated the presence of 1-bp (G) deletion between the third and first nucleotides upstream of the PAM with an efficiency of 20% (2/10) (Table S2; Figure S2B-a). In the case of sample #a-3, of the eight sub-clones examined, two (25%) had a 2-bp (AG or GA) deletion adjacent to the PAM, or between the fourth and first nucleotides upstream of the PAM, and one (13%) had a 1-bp (G to C) replacement of the second nucleotide flanking the PAM (Table S2; Figure S2A-b). Five of the eight clones (63%) examined had a normal sequence (Table S2). As for the whole body sample, 11% (1/9) of the sub-clones had a 2-bp (AG or GA) deletion immediately next to the PAM, and 11% (1/9) had a 1-bp (G to C) replacement involving the second nucleotides adjacent to the PAM (Table S2; Figure S2B-b). These results suggested that similar modes of mutation at the target locus occur in both the fetal heart and the whole body tissues. A similar result was also obtained for the other sub-clones derived from samples #a-4 and #a-7 (Table S2; Figure S2). 

Notably, among the transgene-positive samples (#a-1 to -10; Figure 2; Table S1), the sequences of the target *MHCα* genomic DNA from samples #a-2, -5, -6, and -8 to -10 were normal (Figure 2; Table S1). These results suggested that there might have been no expression of Cas9 or that of the gRNA targeted to *MHCα* genomic DNA in these samples. Alternatively, expression might have been weak, or exhibited low frequency of “indel” detection, which might have contributed to the generation of extremely small numbers of genome-edited cells.

### 3.3. Off-Target Effects

Off-target effects are a serious problem when the CRISPR/Cas9 system is applied to HGD-based TPGD-GEF. We checked possible sites amenable to Cas9 endonuclease-mediated DNA cleavage using a Cas-OFFinder program (http://www.rgenome.net/cas-offinder/). We identified two candidate genes (Figure S3). We prepared each primer set to amplify a target site by PCR, and the resulting PCR products were subjected to direct sequencing. We used four fetal cardiac samples (#a-1, -3, -4, and -7), all of which had been judged to show genome editing (Figure 3; Table S2). Direct DNA sequencing demonstrated that, in all of these samples, no obvious indels were noted for each candidate gene (Figure S3).

## 4. Discussion

In a previously study [11], we focused on the successful uptake of a FuGENE6-encapsulated plasmid pCGSap1-*EGFP* (designed for the targeted disruption of *EGFP* cDNA) injected into the tail vein of pregnant females on E12.5 by fetal cardiac cells, the expression of both Cas9 and gRNA in those cells, and, finally, the induction of mutations in the chromosomally-integrated *EGFP* genomic target sequence. This is based on our previous notion that DNA introduced through TPGD at E12.5 can be preferentially taken up by fetal cardiac cells [13]. Indeed, we found reduced expression of EGFP-derived fluorescence in TPGD-treated hearts, and this event was closely associated with the presence of the *Cas9* gene and mutations in *EGFP* cDNA [11]. Based on this, we next sought to target the endogenous *MHCα* gene [24,25,26], whose dysfunction is known to cause HCM and DCM in humans [27,36]. In this case, we employed another gene delivery approach, termed HGD, which relies on the introduction of a large volume of DNA solution at once and is a powerful method for the efficient transfection of murine hepatocytes [29,30]. Based on the original study [29], this technology used naked DNA and does not require any gene delivery reagents with potential cytotoxicity. The application of HGD to TPGD has been previously reported by two groups [14,19] using plasmid DNA. For example, Wu et al. [14] used naked plasmid or plasmid complexed with PEI for TPGD. They observed successful gene delivery in various tissues of pregnant females and fetuses only when naked DNA complexed with PEI was subjected to HGD-based TPGD. Unlike the approaches taken by the above two groups, we chose to use TransIT-EE, a polyamine-based solution which is commercially available with proven efficacy for transfecting hepatocytes in vivo [31]. Polyamine exists naturally in living organisms and is also found in milk. In this sense, the use of TransIT-EE for HGD-based TPGD-GEF appears to be a novel and safer approach when gene editing is targeted to the fetal genome. Notably, we failed to obtain genome-edited fetuses when conventional TPGD was applied using naked plasmid DNA (pCGSap1-*MHCα*) dissolved in TransIT-EE (data not shown). Efremov et al. [19] reported a similar result (unpublished data) whereby that introduction of naked DNA via conventional TPGD failed to transfect fetuses.

In this study, indel occurrence was observed at the endogenous *MHCα* locus in some of the fetuses carrying pCGSap1-*MHCα,* when HGD-based TPGD-GEF was performed on pregnant females at E9.5. Although the mode of mutation in the obtained fetuses was mosaic (as shown by the presence of edited and unedited cells) (see Figure 3), evidence of genome editing in some of the fetal cells indicated that the uptake and expression of CRISPR/Cas9-related components by fetal cells indeed occurred after HGD-based TPGD-GEF. Details of the nucleotide sequences of the successfully genome-edited fetuses after sub-cloning analysis are summarized in Table S2. Notably, data shown in Table S2 were found to be consistent with those obtained when CRISP-ID analysis was applied using the Sanger sequence chart [37]. In this case, the chromosomal integration of the CRISPR/Cas9 vector is not always required; in other words, transient expression of this vector is sufficient for that purpose, as indicated by Sato et al. [8]. 

The most interesting finding clarified by the present study is that HGD is useful to transfect E9.5 fetal cells, which was shown to be ineffective by our previous method (based on the general (not HGD) tail vein injection of FuGENE6-encapsulated plasmid DNA) [11,13]. According to Kikuchi et al. [13], the plasmid DNA introduced into the tail vein of pregnant mice on E12.5 is preferentially taken up by fetal cardiac cells. They speculated that this might be closely associated with placental establishment, through which exogenous DNA might be delivered to fetuses via the circulatory system. However, as discussed in our previous paper [10], placental development at E9.5 of pregnancy still appears to be poor. Thus, incorporation of plasmid DNA by fetal cells at E9.5 appears to occur via other placenta-independent routes, although the detailed mechanisms involved are still unclear. Furthermore, whole body, as well as the fetal heart tissue, can be successfully genome-edited by our present HGD-based TPGD-GEF (see Figure 3; Table S2). This enables us to investigate the effects of CRISPR/Cas9-based genome editing on various types of cells or tissues (in addition to fetal cardiac cells) in offspring obtained after HGD-based TPGD-GEF. Notably, in agreement with our present findings, Tsukamoto et al. [12], who developed TPGD technology for the first time, reported that the tail vein introduction of a liposome-encapsulated β-actin promoter-based lacZ expression plasmid vector into pregnant female mice at E8.5, using a general method, caused broad expression of lacZ throughout an entire fetus when cells were collected 2 days after gene delivery. 

One of the concerns associated with HGD-based TPGD-GEF is the low success rate (Figure 2). For example, of the three pregnant females tested, only one had Tg fetuses, and the other two had non-Tg fetuses when HGD was performed at E9.5 (Table S1). Similarly, HGD at E12.5 and E15.5 failed to deliver CRISPR/Cas9 components to fetuses (Figure 2; Table S1). Although the reproducibility of the method is important for establishing HGD-based TPGD-GEF, low reproducibility has always been associated with the TPGD system [13]. Probably, this failure may be associated with the establishment of the nascent placental system as discussed previously [10]; however, at present it is not clear how to increase the reproducibility. To gain robust results in a timely manner, we attempted to introduce large amounts of DNA using HGD as a preliminary test. When HGD-based TPGD-GEF was performed by administrating increased amounts of plasmid DNA (60 µg/mouse; n = 2) dissolved in TransIT-EE to pregnant female mice at E9.5, E12.5, and E15.5, both of the females treated at E9.5 had numerous dead fetuses. Under these poor conditions, we were able to collect two fetuses from one female and one fetus from the other female. All these fetuses collected appeared morphologically normal. Only the former two fetuses were found to have introduced pCGSap1-*MHCα* vector, and one of those exhibited genome editing at *MHCα* locus (data not shown). From the females treated at E12.5 and E15.5, several normal-looking fetuses were obtained, but all were judged to be non-Tg (data not shown). We thought that the frequent fetal deaths observed when HGD was performed at E9.5 might be partly caused by applying a large amount (3-fold higher than the amount used in this study) of plasmid DNA. Probably, administration of a large amount of plasmid DNA is toxic to fetuses at early stages of development. As another trial, we are now testing another round of gene delivery reagents (i.e., biodegradable nanoparticles) allowing efficient delivery of NAs (20 µg/mouse) and using increased numbers of pregnant females.

It is also important to explore the factors affecting the low reproducibility in TPGD-GEF. For example, there is a need to examine the livers of pregnant mothers in order to determine the frequency of genome editing in a target gene in that organ. Its frequency may be a useful indicator for predicting the efficiency of HGD-based TPGD-GEF. This may also be needed to examine relationships between the copy number of transgenes introduced into fetuses and the degree of successful editing, since there is a close correlation between fetuses with exogenous DNA and those presenting genome editing at a target locus (see Figure 2; fetuses #a-1 to -10 in Table S1 and Figure S1). A gRNA search, aided by bioinformatics, can provide a high degree of genome editing efficiency, and will be helpful for increasing success in HGD-based TPGD-GEF.

In this study, we detected indels in both the heart and in the “whole body” (which is a part of a fetus except for heart) when fetuses were identified as carrying the pCGSap1-*MHCα* vector (see Figure S1; Tables S1 and S2). This suggests that HGD-based TPGD enables the transfection of all types of fetal cells, just not fetal cardiac cells. The use of mice produced through HGD-based TPGD-GEF in future studies will be important to examine the types of fetal cells transfected by this procedure. Previously, we used *lacZ* gene-floxed Tg mice who were later injected with a Cre expression plasmid [13]. In this way, we found that fetal cardiac cells and other vertebral cells are mainly stained by X-Gal, a substrate for β-galactosidase. If HGD-based TPGD is applied to these *lacZ* gene-floxed mice, the localization of cells with gene switching can be easily monitored. Alternatively, it will be possible to confirm this using mice obtained after HGD-based TPGD, because segregation of internal organs is easy and possible at this stage.

As mentioned previously, HGD-based TPGD-GEF was shown to elicit CRISPR/Cas9-mediated mutations at the endogenous *MHCα* locus in fetal cells (including cardiac cells), although it produced a mixture of both unedited and edited cells. This suggested the possibility of manipulation of embryonic cardiac cells through attenuating the expression of *MHCα* via HGD-based TPGD-GEF. For example, HCM, a disease caused by the expression of mutant MHCα, can be improved, when the level of mutant MHCα is reduced to ~75% in experimental mice [38]. In this context, the present HGD-based TPGD-GEF may be applicable to the production of mouse models for HCM. As inhibition of HCM was achieved by only a 25% reduction in the levels of the mutant transcripts, we suggest that the variable clinical phenotype in HCM patients reflects allelic-specific expression and that partial silencing of mutant transcripts may have therapeutic benefit [38]. A recent study using an RNAi-based knockdown of mutated *MHCα* gene expression made it possible to cure such genetic disorders [38]. In this study, we aimed to create HCM model mice with a genome-edited *MHCα* locus by HGD-based TGD-GEF. This attempt appears to be the first of such, and we believe that our trial will succeed. This is because, theoretically, the generation of HCM is sufficient to achieve this and only 30% of cardiac cells can exhibit a dysfunction in *MHCα* locus. If these model mice are obtained, it may be possible to develop a strategy based on RNAi technology (mentioned above) or knock-in technology for the treatment of such diseases.

HGD-based TPGD-GEF theoretically enables genome editing at multiple target loci of a fetus when appropriate vector(s) are prepared, which enables the expression of multiple gRNAs. If realized, the usability of this system would increase substantially.

## 5. Conclusions

We sought to introduce a single plasmid conferring expression of both Cas9 and gRNA targeted to the endogenous *MHCα* locus into fetuses using HGD-based TPGD-GEF in pregnant females at E9.5, E12.5, and E15.5 of gestation. When the dissected hearts and the other remaining tissues (designated as whole body) were analyzed 2 days after transfection, one of three pregnant mice at E9.5 was found to have Tg fetuses. Of these, 100% (10/10) were found to possess the introduced DNA. Of those, 40% exhibited successful genome editing of the target sequence, although the embryonic cardiac cells comprised a mixture of genome-edited and unedited cells. HGD-based gene transfer failed to transfect fetuses on E12.5 and E15.5. This study has novelty, which is clearly different from other similar research including our previous paper in view that: 1) successful genome editing was achieved when HGD was employed without using any gene delivery reagents, and 2) mutations are induced successfully at the target endogenous *MHCα* locus. In addition, against our expectations, cells with genome editing were found in cardiac cells, and in other non-cardiac cells. E9.5 appears to be the appropriate stage for manipulating the function of embryonic cells by the HGD-based TPGD-GEF, although there is still room for improvements to this system.

## Figures and Tables

**Table 1:** Nucleotide sequences of oligonucleotides (oligos) used in this study.

Oligo Type	Name of Oligo	Sequence (5’–3’)
crRNA	E-oligo-S ^1^	ACC GCA GAA TGA CGG ACG CCC AGA G
crRNA	E-oligo-AS ^1^	AAA CTC TGG GCG TCC GTC ATT CTG C
Primer	Sap1-2S ^2^	TAC AAG GCT GTT AGA GAG ATA
Primer	Sap1-RV ^2^	TCT TAT GGA GAT CCC TCG ACC
Primer	mEx4-S ^3^	GCA AAT GTG GAT GCT GGG AAC
Primer	mEx4-RV ^3^	ACA GTT TTA ATG GCC ATC TGG
Primer	MHC60-1S ^4^	GAG AGC CAT AGG CTA CGG TG
Primer	MHC60-1RV ^4^	CTG TCT TGC CAC CAT TGC AC
Primer	MHC60-2S ^5^	AGG GAA GTG GTG GTG TAG GA
Primer	MHC60-2RV ^5^	ATG TCA AAG GGC CGT GTC TG
Primer	Fyn-S ^6^	GCA AAT GCA GCC ATA TTG GGC
Primer	Fyn-RV ^6^	TCT TGG AGC CAG GGG TAA TGA
Primer	RP24-S ^7^	CCG CAT TGG CAT GGT AGT ACC
Primer	RP24-RV ^7^	TAG AAT TCG GTC CAA CTG CAA

^1^ These oligos were used for synthesis of guide RNA (gRNA) targeting the myosin heavy-chain α (*MHCα*) gene. ^2^ This primer set was expected to yield 435-bp products from the pCGSap1-*MHCα* plasmid (see Figure 1A). ^3^ This primer set was expected to produce 390-bp fragments from the murine endogenous α-1,3-galactosyltransferase (*α-GalT*) sequence. ^4^ This primer set was expected to produce 363-bp fragments from the *MHCα* gene. MHC60-1S was also used for sequencing. ^5^ This primer set was expected to produce 206-bp fragments from *MHCα* by nested PCR. MHC60-2S was also used for sequencing. ^6^ This primer set was used for PCR for off-target analysis. It was expected to produce 401-bp fragments from the murine *Fyn* sequence, located on chromosome 10. Fyn-S was also used for sequencing. ^7^ This primer set was used for PCR for off-target analysis. It was expected to produce 590-bp fragments from the murine DNA sequence from clone RP24-296K22 on chromosome 14. RP24-S was also used for sequencing.

## Supplementary Materials

The following are available online at https://www.mdpi.com/2073-4409/9/7/1744/s1, Table S1: Summary of analysis of fetuses obtained after HGD-based TPGD-GEF on E9.5, 12.5 or 15.5. Table S2: Nucleotide sequences of a region spanning a sequence recognized by gRNA in pCGSap1-*MHCα* in sub-clones from #a-1, #a-3, #a-4, and #a-7 samples. Figure S1: PCR analysis of genomic DNA isolated from fetal samples (#a-1 to -10) derived from recipient #A (Figure 2; Table S1). Figure S2: Sequencing of PCR products derived from the genome-edited fetuses sub-cloned into a pTA cloning vector. Figure S3: Off-target analysis of 2 candidate genes for fetal hearts exhibiting indels.

## Abbreviations

Amp^r^	ampicillin resistance gene
α-GalT	α-1,3-galactosyltransferase
BPB	blood-placental barrier
B6C3F1	mouse hybrid between C57BL/6 and C3H/H
CAG	chicken ß-actin-based promoter
Cas9	clustered regularly interspaced short palindromic repeats (CRISPR)-associated protein-9 nuclease
CRISPR	clustered regularly interspaced short palindromic repeats
E	embryonic day
EGFP	enhanced green fluorescent protein
GGTA1	α-1,3-galactosyltransferase
gRNA	guide RNA
HCM	hypertrophic cardiomyopathy
HGD	hydrodynamics-based gene delivery
lacZ	β-galactosidase
MHCα	myosin heavy-chain α
NAs	nucleic acids
NC	negative control
oligos	oligonucleotides
PAM	protospacer adjacent motif
PC	positive control
PEIs	polyethylenimines
Sry	sex-determining region Y
Tg	transgenic
TPGD	transplacental gene delivery
TPGD-GEF	transplacental gene delivery (TPGD) for acquiring genome-edited fetuses
U6	human *U6* promoter
WT	wild-type
