# Supplementary material for: Hydrodynamics-Based Transplacental Delivery as a Useful Noninvasive Tool for Manipulating Fetal Genome
Source: Cells. 2020 Jul 21;9(7):1744. doi: 10.3390/cells9071744 (PMC7409276; doi:10.3390/cells9071744)
Supplement: Supplementary file 1 [file cells-09-01744-s001.pdf]

Table S1. Summary of analysis of fetuses obtained after HGD-based TPGD-GEF on E9.5, 12.5 or 15.5.

E9.5				E12.5				E15.5			
Name of pregnant females	Name of fetuses	Transgene (pCGSap1-*MHCα*)	Presence of mutations in the target mouse *MHCα*[1]	Name of pregnant females	Name of fetuses	Transgene (pCGSap1-*MHCα*)	Presence of mutations in the target mouse *MHCα*[1]	Name of pregnant females	Name of fetuses	Transgene (pCGSap1-*MHCα*)	Presence of mutations in the target mouse *MHCα*[1]
#A	#a-1	Yes	Mosaic	#D	#d-1	No	Normal	#G	#g-1	No	Normal
	#a-2	Yes	Normal		#d-2	No	Normal		#g-2	No	Normal
	#a-3	Yes	Mosaic		#d-3	No	Normal		#g-3	No	Normal
	#a-4	Yes	Mosaic		#d-4	No	Normal		#g-4	No	Normal
	#a-5	Yes	Normal		#d-5	No	Normal		#g-5	No	Normal
	#a-6	Yes	Normal		#d-6	No	Normal		#g-6	No	Normal
	#a-7	Yes	Mosaic		#d-7	No	Normal		#g-7	No	Normal
	#a-8	Yes	Normal		#d-8	No	Normal		#g-8	No	Normal
	#a-9	Yes	Normal		#d-9	No	Normal		#g-9	No	Normal
	#a-10	Yes	Normal		#d-10	No	Normal		#g-10	No	Normal
#B	#b-1	No	Normal		#d-11	No	Normal		#g-11	No	Normal
	#b-2	No	Normal	#E	#e-1	No	Normal		#g-12	No	Normal
	#b-3	No	Normal		#e-2	No	Normal	#H	#h-1	No	Normal
	#b-4	No	Normal		#e-3	No	Normal		#h-2	No	Normal
	#b-5	No	Normal		#e-4	No	Normal		#h-3	No	Normal
	#b-6	No	Normal		#e-5	No	Normal		#h-4	No	Normal
	#b-7	No	Normal		#e-6	No	Normal	#I	#i-1	No	Normal
	#b-8	No	Normal		#e-7	No	Normal		#i-2	No	Normal
	#b-9	No	Normal		#e-8	No	Normal		#i-3	No	Normal
	#b-10	No	Normal		#e-9	No	Normal		#i-4	No	Normal
	#b-11	No	Normal		#e-10	No	Normal		#i-5	No	Normal
	#b-12	No	Normal		#e-11	No	Normal		#i-6	No	Normal
	#b-13	No	Normal	#F	#f-1	No	Normal		#i-7	No	Normal
	#b-14	No	Normal		#f-2	No	Normal		#i-8	No	Normal
#C	#c-1	No	Normal		#f-3	No	Normal		#i-9	No	Normal
	#c-2	No	Normal		#f-4	No	Normal		#i-10	No	Normal
	#c-3	No	Normal		#f-5	No	Normal		#i-11	No	Normal
	#c-4	No	Normal		#f-6	No	Normal		#i-12	No	Normal
	#c-5	No	Normal		#f-7	No	Normal		#i-13	No	Normal
	#c-6	No	Normal		#f-8	No	Normal		#i-14	No	Normal
	#c-7	No	Normal		#f-9	No	Normal		#i-15	No	Normal
	#c-8	No	Normal						#i-16	No	Normal
									#i-17	No	Normal

[1] Mode of mutations in the murine *MHCα* gene in heart and other parts of a fetus (whole body) was almost the same for each sample.

Table S2. Nucleotide sequences of a region spanning a sequence recognized by gRNA in pCGSap1-*MHCα* in sub-clones from #a-1, #a-3, #a-4, and #a-7 samples [1].

Name of fetuses	Fetal portion examined [2]	Mode of mutations in sub-clones	Sequence (5'-3')/ PAM	Rate
#a-1	Heart	Wild-type	5′- CAGAATGACGGACGCCCAGATGG -3′	6/12 (50%)
		1-bp deletion (-G)	5′- CAGAATGACGGACGCCCA - ATGG -3′	3/12 (25%)
		2-bp deletion (-AG or GA)	5′- CAGAATGACGGACGCCC - - ATGG -3′<br>or<br>5′- CAGAATGACGGACGCCCA - - TGG -3′	2/12 (17%)
		1-bp replacement (G to C)	5′- CAGAATGACGGACGCCCACATGG -3′	1/12 (8%)
	Whole body	Wild Type	5′- CAGAATGACGGACGCCCAGATGG -3′	8/10 (80%)
		1-bp deletion (-G)	5′- CAGAATGACGGACGCCCA - ATGG -3′	2/10 (20%)
#a-3	Heart	Wild-type	5′- CAGAATGACGGACGCCCAGATGG -3′	5/8 (63%)
		2-bp deletion (-AG or GA)	5′- CAGAATGACGGACGCCC - - ATGG -3′<br>or<br>5′- CAGAATGACGGACGCCCA - - TGG -3′	2/8 (25%)
		1-bp replacement (G to C)	5′- CAGAATGACGGACGCCCACATGG -3′	1/8 (13%)
	Whole body	Wild-type	5′- CAGAATGACGGACGCCCAGATGG -3′	7/9 (78%)
		2-bp deletion (-AG or GA)	5′- CAGAATGACGGACGCCC - - ATGG -3′<br>or<br>5′- CAGAATGACGGACGCCCA - - TGG -3′	1/9 (11%)
		1-bp replacement (G to C)	5′- CAGAATGACGGACGCCCACATGG -3′	1/9 (11%)
#a-4	Heart	Wild-type	5′- CAGAATGACGGACGCCCAGATGG -3′	8/14 (57%)
		1-bp deletion (-A)	5′- CAGAATGACGGACGCCCAG - TGG -3′	1/14 (7%)
		1-bp deletion (-C)	5′- CAGAATGACGGACGCC - AGATGG -3′<br>or<br>5′- CAGAATGACGGACGC - CAGATGG -3′<br>or<br>5′- CAGAATGACGGACG - CCAGATGG -3′	1/14 (7%)
		1-bp deletion (-G)	5′- CAGAATGACGGACGCCCA - ATGG -3′	2/14 (14%)
		2-bp deletion (-AG or GA)	5′- CAGAATGACGGACGCCC - - ATGG -3′<br>or<br>5′- CAGAATGACGGACGCCCA - - TGG -3′	2/14 (14%)
	Whole body	Wild-type	5′- CAGAATGACGGACGCCCAGATGG -3′	10/13 (77%)
		1-bp deletion (-A)	5′- CAGAATGACGGACGCCCAG - TGG -3′	1/13 (8%)
		1-bp deletion (-C)	5′- CAGAATGACGGACGCC - AGATGG -3′<br>or<br>5′- CAGAATGACGGACGC - CAGATGG -3′<br>or<br>5′- CAGAATGACGGACG - CCAGATGG -3′	1/13 (8%)
		1-bp deletion (-G)	5′- CAGAATGACGGACGCCCA - ATGG -3′	1/13 (8%)
#a-7	Heart	Wild-type	5′- CAGAATGACGGACGCCCAGATGG -3′	6/11 (55%)
		1-bp deletion (-A)	5′- CAGAATGACGGACGCCCAG - TGG -3′	2/11 (18%)
		1-bp deletion (-G)	5′- CAGAATGACGGACGCCCA - ATGG -3′	3/11 (27%)
	Whole body	Wild-type	5′- CAGAATGACGGACGCCCAGATGG -3′	10/13 (77%)
		1-bp deletion (-A)	5′- CAGAATGACGGACGCCCAG - TGG -3′	1/13 (8%)
		1-bp deletion (-G)	5′- CAGAATGACGGACGCCCA - ATGG -3′	2/13 (15%)

[1] Fetal DNA was PCR-amplified using primers corresponding to murine *MHCα* gene exon 1. The resultant PCR products were then sub-cloned into a pTA cloning vector. Sequencing results of the inserts sub-cloned are shown.

[2] Fetuses were divided into two portions [heart and the other part of a fetus (whole body)], prior to molecular biological analysis.

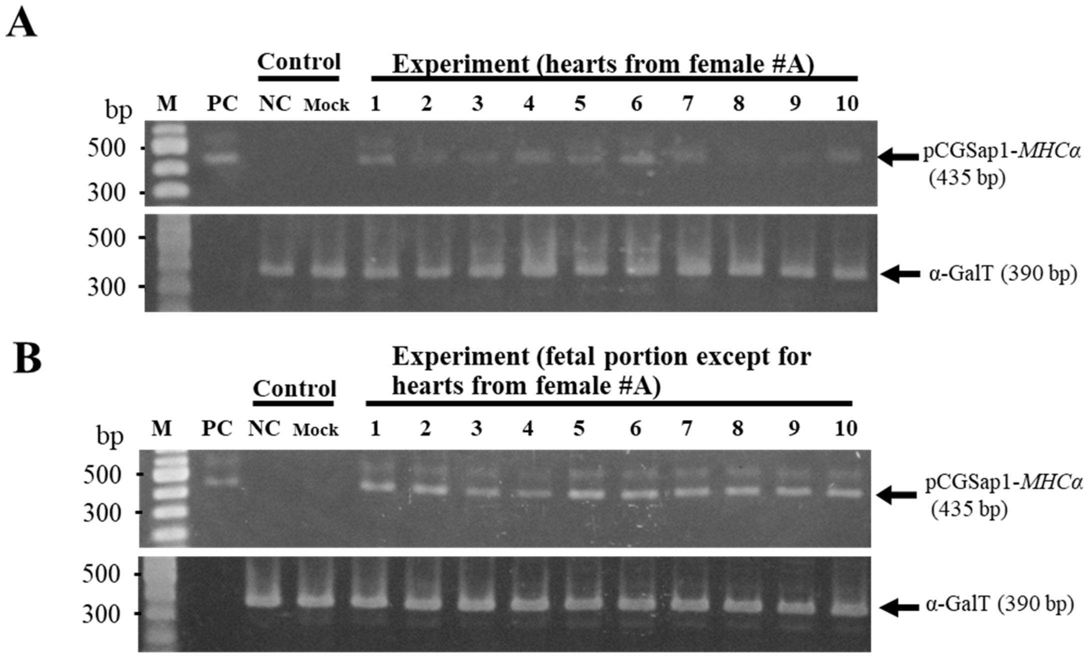

Figure S1. PCR analysis of genomic DNA isolated from fetal samples (#a-1 to -10) derived from recipient #A (Figure 2; Table S1). **A**. PCR analysis of fetal hearts. Note the presence of the transgene (pCGSap1-*MHCα*) in all of the samples tested, when a primer set Sap1-2S/Sap1-RV (see Figure 1A) recognizing a region spanning the gRNA-binding sequence was used for PCR. α-GalT, α-1,3-galactosyltransferae gene; PC, positive control (5 ng of pCGSap1-*MHCα*) was used; NC, negative control [genomic DNA (approximately 5 ng) from non-Tg tail tissue]; Mock, genomic DNA (approximately 5 ng) from mock-injected fetal heart; M, 100-bp ladder markers. **B**. PCR analysis of whole body (fetal portion except for heart). PCR and gel electrophoresis of the PCR products are the same shown in **A**.

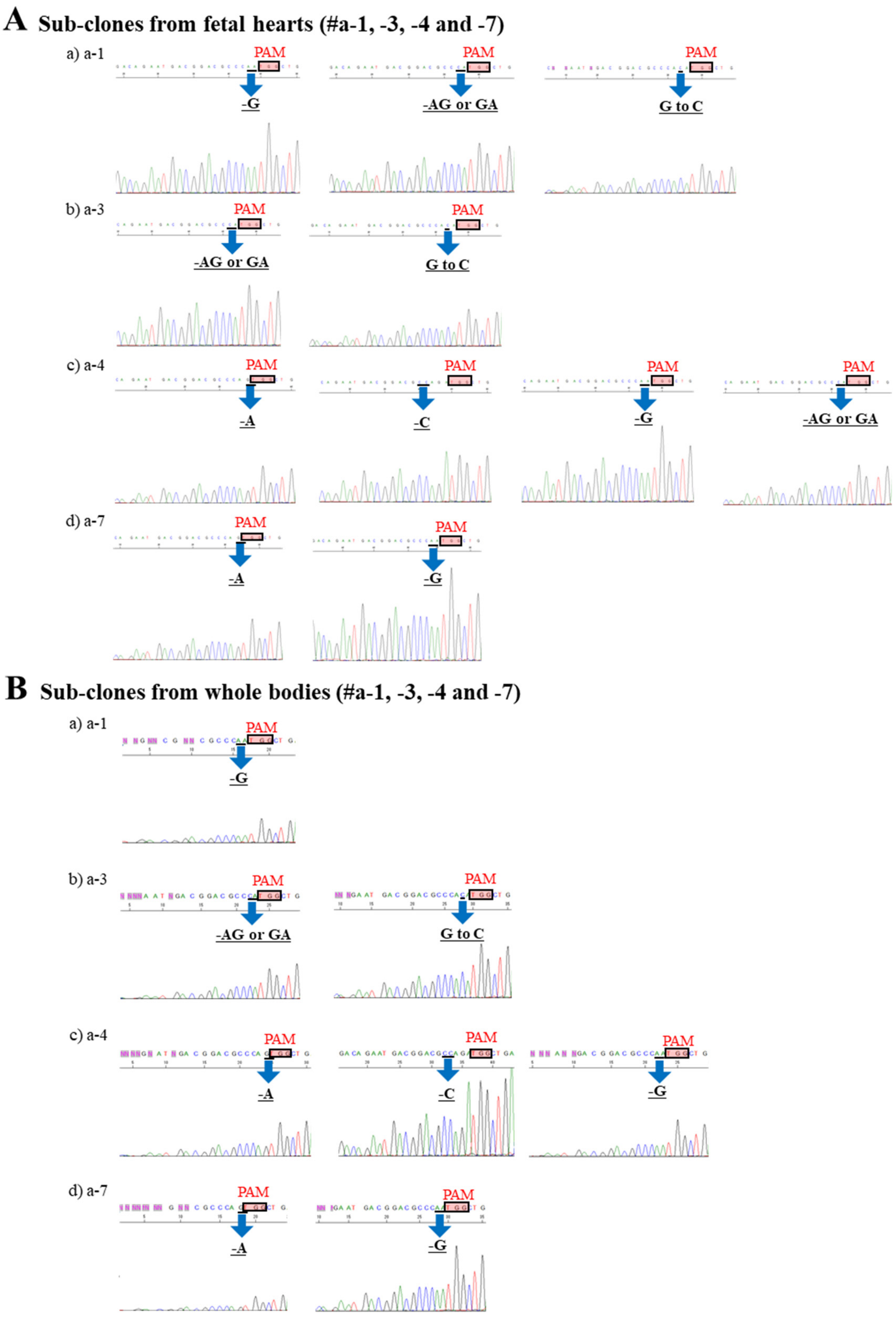

Figure S2. Sequencing of PCR products (corresponding to the ATG-containing region of *MHCα* recognized by gRNA) derived from genome-edited fetuses sub-cloned into a pTA cloning vector. **A**. Direct sequencing of PCR products obtained from fetal hearts (#a-1, -3, -4, and -7). In each case, indels are notable immediately upstream of the PAM (TGG) (shown in red). **B**. Direct sequencing of PCR products obtained from the whole bodies (#a-1, -3, -4, and -7). In each case, indels are notable immediately upstream of the PAM (shown in red).

A chr10 39458123 (CAaAATGAaGGACCAGCCCAGATGG): Mus musculus strain C57BL/6J chromosome 10, GRCm38.p6 C57BL/6J; NCBI Reference Sequence: NC_000076.6

The target sequence was PCR-amplified using Fyn-S/Fyn-RV primer set (see Table 1), and the resulting PCR products are subjected to direct sequencing using Fyn-S.

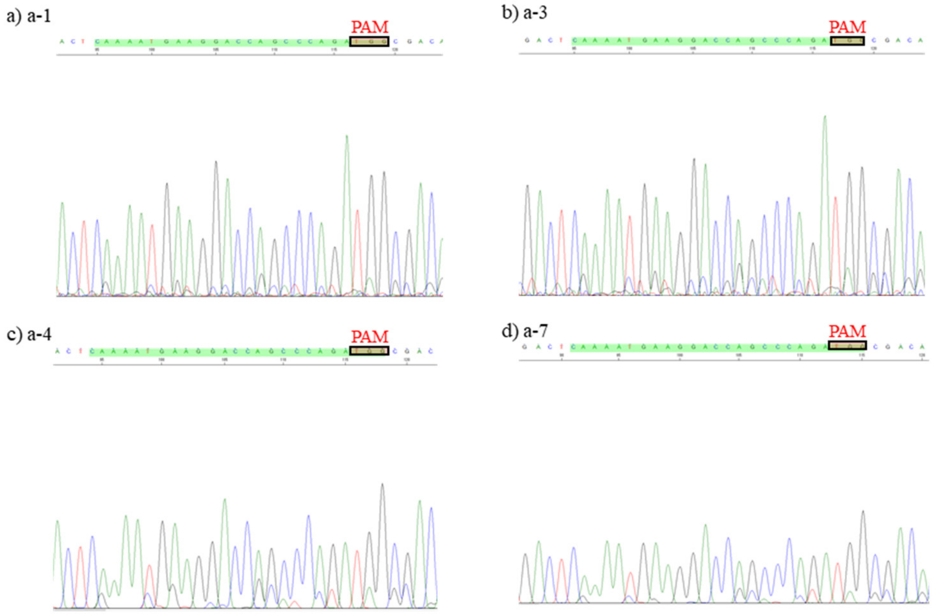

B chr14 55584929 (CAGcATGACGGACAAGgCCAGATGG): Mouse DNA sequence from clone RP24-296K22 on chromosome 14, complete sequence; Sequence ID: CT025679.5Length: 160805Number of Matches: 1

The target sequence was PCR-amplified using RP24-S/RP24-RV primer set (see Table 1), and the resulting PCR products are subjected to direct sequencing using RP24-S.

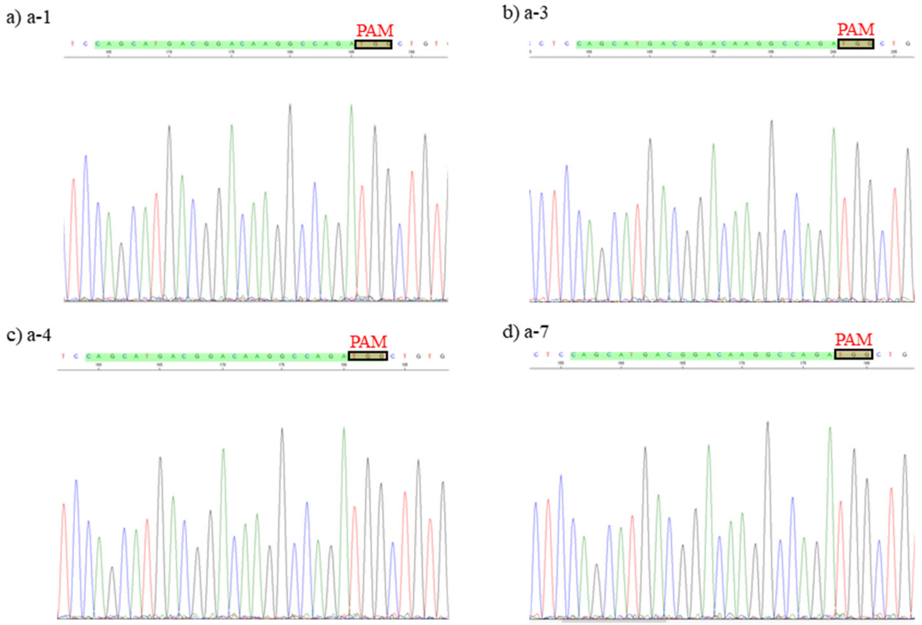

Figure S3. Off-target analysis of 2 candidate genes for fetal hearts (#a-1, -3, -4, and -7) exhibiting indels. Genomic DNA isolated from fetal hearts was PCR-amplified using a primer set for each candidate gene (Table 1). Direct sequencing of the resulting PCR products was performed using a sense primer.
